# Rheumatoid Arthritis and Primary Biliary Cirrhosis: Cause, Consequence, or Coincidence?

**DOI:** 10.1155/2012/391567

**Published:** 2012-10-24

**Authors:** Daniel S. Smyk, Dimitrios P. Bogdanos, Maria G. Mytilinaiou, Andrew K. Burroughs, Eirini I. Rigopoulou

**Affiliations:** ^1^Institute of Liver Studies, Division of Transplantation Immunology and Mucosal Biology, School of Medicine, King's College London, London SE5 9RS, UK; ^2^Department of Medicine, University Hospital of Larissa, University of Thessaly Medical School, Viopolis, 41110 Larissa, Greece; ^3^The Sheila Sherlock Liver Centre and University Department of Surgery, Royal Free Hospital, London NW32QG, UK

## Abstract

Primary biliary cirrhosis (PBC) is a progressive cholestatic liver disease characterized serologically by cholestasis and the presence of high-titre antimitochondrial antibodies and histologically by chronic nonsuppurative cholangitis and granulomata. PBC patients often have concomitant autoimmune diseases, including arthropathies. This raises the question as to whether there are shared features in the pathogenesis of those diseases with the pathogenesis of PBC. Epidemiological and large case studies have indicated that although the incidence of rheumatoid arthritis (RA) is not significantly raised in PBC patients, there appears to be a higher rate of RA in PBC patients and their relatives. Genetic studies have demonstrated that several genes implicated in PBC have also been implicated in RA. Epigenetic studies provided a wealth of data regarding RA, but the findings on epigenetic changes in PBC are very limited. As well, certain infectious agents identified in the pathogenesis of PBC may also play a role in the pathogenesis of RA. These data suggest that although RA is not significantly present in PBC, some individuals with certain genetic traits and environmental exposures may develop both conditions. This concept may also apply to other concomitant diseases found in PBC patients.

## 1. Introduction

Primary biliary cirrhosis (PBC) is a chronic cholestatic, autoimmune liver disease characterised by progressive inflammatory destruction of the intrahepatic bile ducts, with fibrosis leading to cirrhosis [1–5] and liver failure [6, 7]. The disease most commonly affects middle-aged females [8–10]. PBC is often found to affect more than one member of the same family, and it appears that first degree relatives (FDRs) of PBC patients have and increased risk of developing the disease [1–5]. There is a consensus that the incidence and prevalence of PBC is increasing [11–15]. This may be due to a true increase in the disease or due to the awareness for the disease amongst clinicians and the meticulous diagnostic assessment, such as disease-specific autoantibody testing.

PBC is characterised by several disease-specific autoantibody profiles [16], which are a key component of the diagnostic workup. Included in the autoantibody profiles are antimitochondrial antibodies (AMA) [17, 18] and/or disease-specific antinuclear antibody (ANA) [19, 20]. AMA positivity appears to be indicative of future PBC development [21]. Autoimmune rheumatic diseases such as Sjögren's syndrome and systemic sclerosis, as well as other extra-hepatic autoimmune manifestations such as autoimmune thyroiditis, are frequently found to be concomitant with PBC [2, 22, 23]. PBC generally progresses slowly, although the clinical course in some cases may advance in a fast pace [6, 24]. 

The diagnostic criteria of PBC include (1) biochemical evidence of cholestasis such as elevated alkaline phosphatase (ALP) and *γ*GT, (2) presence of disease-specific antimitochondrial antibodies, and (3) histological features of PBC [2]. Currently, two of the three criteria are needed for PBC diagnosis [2, 4]. Additionally, it is common to see elevated levels of immunoglobulin M (IgM) [2, 4, 25].

PBC-specific AMA predominantly targets the E2 subunits of the oxoacid dehydrogenase complexes (OADCs), and especially that of the pyruvate dehydrogenase complex (PDC-E2) [18, 21, 26]. Approximately 3–10% of patients with PBC are negative for these autoantibodies [21, 27–30]. Although these autoantibodies may be found in other conditions, anti-PDC-E2-specific AMA are rarely found in other conditions [31, 32]. AMA seropositivity in patients with autoimmune rheumatic diseases indicates the presence of PBC or the future development of the disease [33]. Prospective studies have demonstrated that the presence of AMA predicts the future development of PBC in asymptomatic, cholestatic, or acholestatic individuals [17, 34]. Approximately 50% of patients have disease-specific ANA [35–37]. Although AMA does not appear to have prognostic significance, it has been suggested that PBC-specific ANA reactivities may have prognostic significance, although larger studies are needed to substantiate the clinical relevance of these autoantibodies [20, 37–39]. Other autoantibody profiles have also been reported in patients with PBC but their pathogenic relevance and diagnostic utility remain unclear [40–42]. 

Both genetic and environmental triggers have been considered important for the induction of PBC, as well as other autoimmune diseases [43, 44]. Environmental triggers include infectious and noninfectious agents [22, 45–47]. Impairment in the immunosuppressory functions of the host appears to be a feature of PBC [48, 49]. 

As many patients with PBC also demonstrate concomitant rheumatic conditions, this paper will examine the epidemiological and genetic data surrounding rheumatoid arthritis (RA) and PBC, using larger cohort studies and epidemiological studies, as well as genetic investigations. These data may indicate that the presence of RA with PBC and *vice versa* may be due to a causal link, as opposed to a casual observation. A possible link with common infectious triggers will also be introduced. This paper is not intended as a literature review of RA in PBC, but rather as an examination of the presence of the two conditions in one another, as well as common genetic traits that may infer susceptibility to both. These commonalities, and the coexistence of the two conditions in one individual, may contribute to the development of what is known as the Kaleidoscope of autoimmunity [50–57]. This Kaleidoscope highlights the fact that many patients with one autoimmune condition also have other concomitant autoimmune conditions and that patients with one autoimmune disease are at risk of developing another [51, 57]. 

## 2. Rheumatoid Arthritis in Primary Biliary Cirrhosis and *Vice Versa*

Several musculoskeletal conditions are known to affect PBC patients, including hypertrophic osteoarthropathy, osteoporosis, avascular necrosis, medullary bone defects due to cholesterol deposition, and RA [58]. However, it is unclear how many cases fit the diagnostic criteria for RA [58–62]. An early study by Sherlock and Scheuer [63] notes that 5% of a cohort of 100 PBC patients had concomitant RA, with approximately 50% of PBC patients being positive for rheumatoid factor (RF). A study by Siegel and colleagues [58] identified 25 patients who did fit the diagnostic criterion for both RA and PBC. Elevated transaminases, ALP, and bilirubin were observed in the cohort, and 13 patients were in PBC stages 1 or 2 [58]. That study found that RA was diagnosed before PBC in 17 cases (mean 11.8 years prior) [58]. In the remaining cases, RA was diagnosed after PBC, with a mean diagnosis at 5 years after PBC, although this ranged from 2 to 14 years [58]. Those researchers suggested that AMA testing should be performed in RA patients who display abnormal LFTs [58]. Caramella et al. [64] report two cases of RA and PBC with Sjögren's ruled out, where one patient developed PBC before RA and the other developed RA before PBC. That study estimated the prevalence of RA in PBC to be between 1.8 and 5.6%, based on other reports [64–66], but it is unknown whether earlier studies based their RA diagnoses on the American College of Rheumatology (ACR) diagnostic protocols. Marasini and colleagues [66] screened 170 patients with histologically confirmed PBC for connective tissue diseases and Raynaud's phenomenon. Connective tissue disease was found in 47 patients, with 21 having systemic sclerosis, 34 being ANA positive, 33 being ACA positive, and 27 patients having extractable nuclear antigen [66]. Three patients had RA, which was diagnosed using ACR criteria [66]. 

Several other studies have reported the presence of PBC-related features in patients with RA PBC but a clear definition of the hierarchy of events leading to the development of abnormal liver biochemistry with or without features of overt liver disease related to PBC is lacking. Generally, liver disease is not seen as a significant feature of RA, although 18–50% of RA patients have abnormal LFTs [67]. As well, several studies have noted liver pathology in RA patients, and others have examined the presence of PBC in RA patients. One retrospective autopsy series of RA patients prior to methotrexate use found that 65% of 182 RA cases with liver biopsies had evident liver pathology [68]. This included chronic mild inflammatory infiltrates of portal tracts and small foci of necrosis, as well as steatosis [68]. However, it is not known whether any of those patients had diagnosed PBC. Another study notes that AMA positivity in RA at 18% [69]. This is of interest given the predictive value of AMA for PBC, although the specificity of the AMA in RA patients is unclear. 

The above studies indicate that RA is present in some PBC patients and *vice versa*, but larger *prospective* studies are needed to determine whether there is a significant and undisputed evidence of the presence of one disease in the other. As well, genetic studies may also shed light on the predisposition of some individuals to develop both conditions. 

## 3. The Link of PBC with RA: Is There Any Evidence from Epidemiological Studies?

 Several epidemiological studies have been conducted to investigate risk factors for the development of PBC [70–73]. These studies have largely been based on questionnaires that investigate geographical and lifestyle data, in addition to personal and familial medical and surgical histories. A study by Parikh-Patel and colleagues [72] administered a standardized US national Health and Nutrition Examination Study (NHANES) questionnaire to 241 PBC patients from the USA, in addition to 261 of their siblings and 141 friends as controls. No mention was made as to the incidence of RA in the PBC cohort [72]. Another study conducted by Gershwin et al. [71] also utilised a NHANES style questionnaire. The cohort in that study consisted of 1032 PBC patients from 23 tertiary care centres in the USA, 1041 controls selected from a random-digit-dialling protocol, which were sex, age, race and geographically matched. In the medical histories, it was found that 10% of PBC cases also had RA, although 8% of controls also reported RA [71]. RA was reported in 26% of first degree relatives (FDRs) of PBC patients, compared to 22% of control relatives [71]. Interestingly, among the FDR, females reported a higher incidence of RA than the male relatives. Mothers of PBC patients were the most affected (13.8%) followed by sisters (11%) [71]. Prince et al. [73] conducted an epidemiological study involving 318 patients from a geographically defined epidemiological study and 2258 from a PBC support group, in addition to 2438 controls. The odds ratio for RA in the epidemiological group was 1.52 compared to 1.21 in the support group [73]. Corpechot and colleagues [70] administered a standardized questionnaire to 222 PBC patients and 509 age, sex, and residentially matched controls [70]. RA was found in 3% of cases and 1% of controls, which was not found to be statistically significant [70]. As well, 2% of FDR of PBC cases, and 1% of FDR of controls also reported RA, and this was also not found to be statistically significant [70]. Epidemiologically, it does not appear that RA is significantly increased in PBC patients. However, the rates of RA were higher in PBC cohorts as well as female FDR, which may suggest a common background for both conditions in some cases. 

## 4. Genetic Studies in PBC and RA: The Missing Link?

Genetic and genome-wide association studies (GWAS) have recently shed light on the genetic background of PBC and RA. Genes implicated in PBC lay within both HLA and non-HLA regions (Table 1) [74–81]. Genetic and GWA studies have also been performed in RA, and multiple susceptibility loci have been identified, also within HLA and non-HLA regions [82–87]. Interestingly, several common genes have been identified in RA and PBC (Table 1), although a vast majority of implicated genes in both diseases do not overlap. Overlapping genes include HLA-DQB1, STAT4, IRF5, MMEL1, CTLA4, and possibly CXCR5. It may be the case that individuals with a common genetic profile may be more susceptible to developing concomitant RA and PBC, or PBC in RA. On the other hand, some individuals with other genetic characteristics for RA or PBC, in which genes do not overlap, will not be susceptible to developing particular concomitant autoimmune disease. This is likely the scenario in the majority of patients, as the epidemiological data does not suggest a large proportion of RA patients with PBC and *vice versa*. 

## 5. Epigenetic Studies in RA and PBC

An increasing body of evidence accumulated in recent years suggesting that epigenetic changes such as histone modifications and DNA methylation can manipulate immune reactions and participate to the development of auto-aggression [88, 89]. Epigenomic modifications have been demonstrated in systemic lupus erythematosus, Sjögren's syndrome, multiple sclerosis, inflammatory bowel diseases, and several other autoimmune diseases [88, 89]. While the role of epigenetic changes involved in the pathogenesis of RA has been extensively studied, data on the involvement of epigenetics in the induction of PBC are scarce [90, 91]. Evidence of enhanced histone acetylation at promoters of genes pathogenetically-related to RA has been provided [92, 93]. As well, various inhibitors of histone deacetylases have shown promising results in experimental models of RA [94, 95]. Histone deacetylases are the group of enzymes that remove acetyl groups from histone tails, the removal of which condensation of chromatin structures and repression of gene expression [91]. In addition, data obtained by independent groups of investigators have reported evidence of disturbed patterns of DNA methylation in RA [91]. A meticulous analysis of epigenetic changes in RA synovial fibroblasts (RASFs) is underway, and it is anticipated that in the years to come we will delineate the behaviour of RASFs and the exact time frame that the epigenetic changes in RASFs are induced [91]. RASFs respond to proinflammatory cytokines such as interleukin-1*β* and tumor necrosis factor-*α* and can drive joint inflammation [91]. Intriguing new data have demonstrated that RASFs can migrate *via* the bloodstream to implanted cartilage at a distant site; such a migratory potential may explain the polyarticular involvement characteristic of RA [96]. 

In contrast to the wealth of data in RA, epigenetic studies in PBC have been largely studied by one group and the data are limited [90]. Mitchell et al. [90] have tested the hypothesis that X-linked promoters of variable X chromosome inactivation (XCI) genes are dysregulated in PBC through aberrations in promoter methylation. They have based their hypothesis on previous observations indicating that women with PBC are characterized by an enhanced rate of preferential X monosomy, with random XCI in peripheral blood lymphocytes isolated from women with PBC compared to demographically matched women [97]. These investigators have tested their hypothesis in discordant monozygotic twin PBC pairs and found that two genes, *CLIC2 *and *PIN4*, exhibited decreased transcription in 3 out of 4 affected pairs compared to unaffected healthy twins in discordant twin pairs [90]. Of relevance, Chabchoub et al. analyzed XCI profiles of females RA patients and found skewed XCI in 34.2% of them compared to 11% found in the control population [98]. 

## 6. An Infectious Agent Linking PBC and RA

 Assuming that a common genetic link exists between some RA/PBC patients, it may also be the case that common infectious triggers may also be involved in the induction of both diseases in particular individuals. One of the most widely studied infectious triggers of PBC is *Escherichia coli *(*E. coli*), due to the high incidence of recurrent urinary tract infections (rUTI) in PBC patients and the fact that *E. coli* is the most commonly isolated bacteria in rUTI [47]. Molecular mimicry and cross-reactivity between self- and bacterial antigens are believed to play a role in the induction of PBC [99]. Several infections have also been linked to RA, most notably Epstein-Barr's virus, parvovirus B19, chronic hepatitis C virus, *Proteus mirabilis*, *Klebsiella pneumoniae*, and *E. coli* [100, 101]. Of interest, anti-*E. coli* IgM has been found to be elevated in RF-positive RA patients [102]. A recent study by Newkirk and colleagues [100] examined the antibacterial antibody levels in patients with RF-positive and negative inflammatory arthritis. Bacteria were isolated from stool and urine samples, with IgM and IgA antibacterial, and RF antibodies being assessed by ELISA. Increased colonization of Group D *E. coli* was found in RF-positive patients, and increased colonization of Group B2 *E. coli* was found in RF-negative patients [100]. RF-negative patients were noted to have a less severe disease phenotype, but had higher levels of IgA anti-*E. coli* [100]. Those investigators suggest a possible role for *E. coli* in the early pathogenesis of RA [100]. Whether or not *E. coli* plays a significant role in the pathogenesis of RA warrants further investigation. It would be of interest to investigate the incidence of rUTI in PBC patients with RA, as *E. coli* infection compounded with a particular genetic trait may be the missing link between RA in PBC. 

## 7. Conclusion

Concomitant arthritides are observed in patients with PBC, which raises the possibility of a common aetiology between PBC and RA. Indeed, RA is observed in PBC patients and *vice versa*, although not at a significantly increased level. Although epidemiological data of RA in PBC do not suggest a significantly increased incidence of RA in PBC, RA does appear to be a feature in a small number of PBC patients and their relatives. Recent genetic studies have identified several common disease-associated genes between the two conditions, which may infer a susceptibility to both diseases in a minority of patients. Epigenetic modifications are well studied in RA, and such investigations are also needed in PBC. The role of infectious triggers, and especially those responsible for recurrent or complicated urinary tract infections, may serve as a trigger of both conditions, and this warrants further investigation.

## Figures and Tables

**Table 1:** Major susceptibility genes associated with primary biliary cirrhosis (PBC), as reported through genome-wide association studies. Note that several positive associations are shared between PBC and rheumatoid arthritis (RA).

Gene	PBC	RA
HLA		
DR8	+	−
DQB1	+	+
DRB1	+	−
DQA1	+	−
DQA2	+	−
Non-HLA		
STAT4	+	+
SPIB	+	−
IRF5	+	+
IL12A	+	−
IL12RB	+	−
MMEL1	+	+
CXCR5	+	?
NFKB1	+	−
CTLA4	+	+

?: question mark indicates that there are no conclusive data regarding the role of CCXR5 as a risk factor in RA, with some reports providing data in support and other against.

## Abbreviations

AMA: Antimitochondrial antibodies
ANA: Antinuclear antibodies
*E. coli*: *Escherichia coli*
FDR: First degree relative
GWAS: Genome-wide association study
PBC: Primary biliary cirrhosis
PDC-E2: Pyruvate dehydrogenase complex E2 subunit
RA: Rheumatoid arthritis
RF: Rheumatoid factor
RASF: RA synovial fibroblasts
rUTI: Recurrent urinary tract infection
XCI: X chromosome inactivation.
